# Proteomic Profiling Skin Mucus of European Eel *Anguilla anguilla* Infected with Anguillid Herpesvirus

**DOI:** 10.3390/ijms231911283

**Published:** 2022-09-24

**Authors:** Ying-Ying Li, Jin-Xian Yang, Xi Chen, Qiang Chen, Tie-Ying Song, Jun-Qing Ge

**Affiliations:** Institute of Biotechnology, Fujian Academy of Agricultural Sciences, Fuzhou 350003, China

**Keywords:** *Anguilla anguilla*, Anguillid herpesvirus, TMT-labelled, proteomic, skin mucus

## Abstract

Anguillid herpesvirus 1 (AngHV) is an important viral pathogen affecting eel. This study was designed to investigate the potential molecular mechanisms and immune response elicited at the protein levels in the skin mucus of AngHV-infected *Anguilla anguilla*. Tandem mass tag (TMT)-labelling proteomics with the liquid chromatography tandem mass spectrometry (LC-MS/MS) was used for performing quantitative identification of the proteins. In addition, the quantitative protein amount was detected by parallel reaction monitoring (PRM) analysis. A total of 3486 proteins were identified, of which 2935 were quantified. When a protein fold change was greater than 1.3 or less than 0.76, it indicated a differentially expressed protein (DEP). Overall, 187 up-regulated proteins and 126 down-regulated proteins were detected, and most of the DEPs were enriched in the CAMs pathway, intestinal immune pathway, herpes simplex virus 1 infection pathway, phagosome pathway and p53 signaling pathway. The results of the DEPs detected by PRM were highly consistent with the results of the TMT-labelled quantitative proteomic analysis. The findings of this study provide an important research basis for further understanding the pathogenesis of AngHV.

## 1. Introduction

Anguillid herpesvirus 1 (AngHV) is the primary viral pathogen of cultured eel and acts as an important factor causing the decline of wild eel [1,2]. AngHV was initially isolated from *A. japonica* in 1990 by Sano et al. [3]. Since then, AngHV has been widely isolated from cultured eel species in the different regions of the world [4,5,6,7,8]. Serological and genomic sequencing analysis has showed that the isolated AngHV strains from Asia and Europe were identical viral species [4,9,10]. AngHV belongs to *Herpesvirales*, *Alloherpesviridae* and *Cyprinivirus* and has linear double-stranded DNA genome with a total length of 248 kb. It can encode 136 open reading frames (ORFs) [11]. Genome sequence alignment analysis has revealed that AngHV only bears 19~58% identity with other viral members of *Alloherpesviridae* [11]. In addition, a prior study related to the biological and physicochemical properties of AngHV indicated that AngHV has a strong host infection specificity [12]. 

China has the largest eel aquaculture industry in the world, which accounts for approximately 70% of the world’s production. Fujian and Guangdong province are the main breeding areas, and the main breeding species are those of *A. anguilla*, *A. rostrata* and *A. japonica*. “Mucus sloughing and hemorrhagic septicemia disease” is the most harmful infectious disease in juvenile eels of *A. anguilla* and *A. rostrata*, which can lead to significantly high morbidity as well as cumulative mortality and can cause huge economic losses to farmers. Epidemiological analysis has indicated that the disease is primarily caused by viral pathogens. Moreover, in previous studies, we detected AngHV from diseased eel samples using the established PCR detection method [13] and successfully isolated AngHV by using eel ovary cell lines (EO) [14]. Furthermore, the viral challenge assay confirmed that AngHV was the main pathogen of eel “mucus sloughing and hemorrhagic septicemia disease” [15].

Most herpes viruses tend to invade mucosa tissues of the host. For example, Herpes simplex virus type I (HSV-1) infection can cause herpes keratitis [16], whereas Cyprinid herpesvirus 1 (CyHV-1) can cause pathological changes in the eye mucosa of the host [17], and Cyprinid herpesvirus 3 (CyHV-3) can cause gill lesions of the host [18]. Eel “mucus sloughing and hemorrhagic septicemia disease” often begins with the typical clinical symptoms of mucus sloughing, and high levels of AngHV can be detected in the skin mucus, which suggests that AngHV might preferentially invade the cells in the mucus of the host.

The skin mucus of fish can effectively protect the body from pathogen infection and plays an important role in the regulation of the immune system of fish [19]. Fish mucosa contains lysozyme, protease and reactive oxygen free radicals [20,21] and can activate acquired immunity independently, including the various processes of antigen uptake, presentation and antibody secretion [22,23]. TMT/iTRAQ-labelling proteomics with liquid chromatography tandem mass spectrometry (LC-MS/MS) can facilitate analysis of the various biological processes and aid in the elucidation of molecular mechanisms [24,25]; it has been widely used to study pathogen and aquatic animal interactions [26,27,28,29].

In this study, TMT-labelled quantitative proteomic analysis was used to analyze the various proteins expressed in the skin mucus of AngHV-infected *A. anguilla*. The results indicated that the expression levels of several immune-related proteins in the skin mucus of AngHV-infected *A. anguilla* were modulated and thus provide important information for elucidation the pathogenesis of AngHV and the defense mechanisms of infected eels.

## 2. Results

### 2.1. Morphological and Histopathological Changes in the Skin of AngHV-Infected Eels

**Figure 1:**
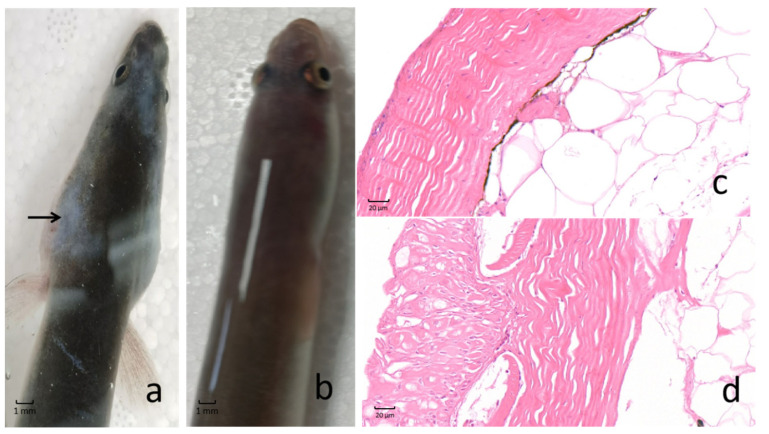
The morphological and histopathological changes observed in the skin in AngHV-infected *A. anguilla*. The morphological image for the eel infected by AngHV (**a**) and the healthy eel (**b**) showed that the skin mucus was sloughed. The histopathological images for the AngHV group (**c**) and the control group (**d**) show that the skin had exfoliation of mucus and mucosa flat epithelial cells in the eel infected by AngHV.

Juvenile European eels were infected with AngHV. At the 6th day post infection, the skin mucus of the eels in the AngHV group displayed exfoliation (Figure 1a), but those in the control group had no obvious clinical changes (Figure 1b). The histopathological analysis of the skin confirmed that AngHV-infected eels possessed distinct exfoliation of skin mucus and had exfoliated skin mucosa comprising the flat epithelial cells (Figure 1c), whereas there were no obvious pathological changes observed in the skin of control eels (Figure 1d).

### 2.2. Protein Identification

**Figure 2:**
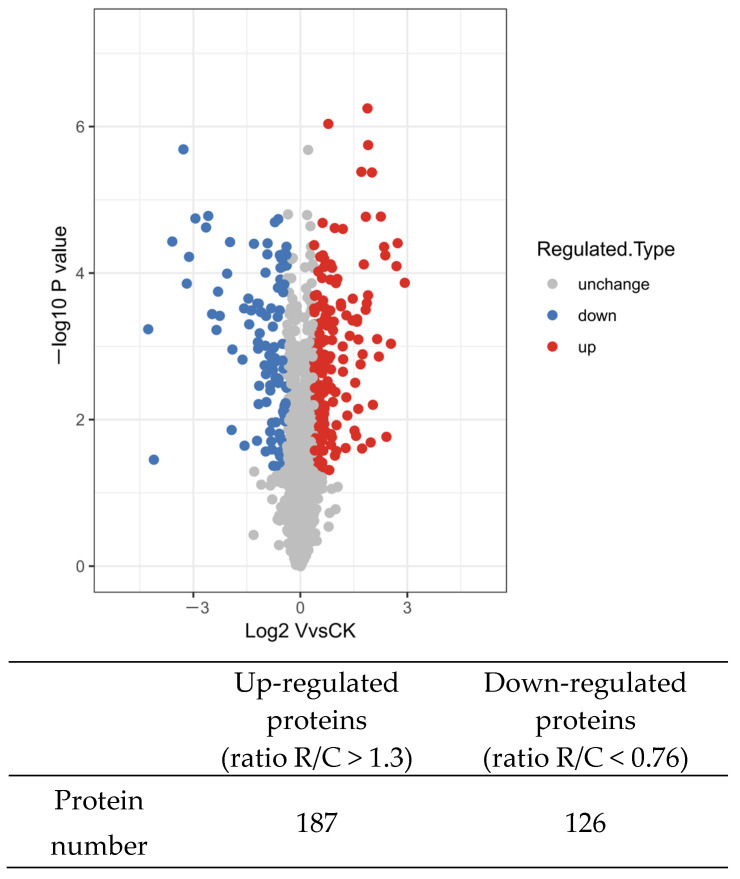
The volcano plot of the DEPs showing both up-regulated (red) and down-regulated (blue) proteins.

The skin mucus of AngHV-infected and control eels was extracted for TMT-based quantitative proteomic analysis. As an important intermediate part of quality control, partial of peptides were selected for single probe labelling efficiency detection; the results indicated that the TMT labelling efficiency reached 99%. A total of 298,207 spectra were detected by TMT analysis, among which 44,795 spectrum were matched. A further database search indicated that 20,633 peptides were obtained, and 16,815 unique peptides were identified. Finally, 3486 proteins were detected, among which 2935 proteins were quantifiable (Table 1); the annotation of the identified proteins were listed in Table S1. The repeatability of the samples in the two groups was good, and the difference between the groups was significant (Figure S1). When a protein fold change was greater than 1.3 or less than 0.76, a total of 313 proteins were detected as differentially expressed proteins (DEPs) (Table S2), including 187 up-regulated proteins and 126 down-regulated proteins (Figure 2).

### 2.3. Functional Classification of the DEPs

In order to analyze the functions and categories of the DEPs, the protein functional annotations were performed from multiple aspects, including Gene Ontology (GO) classification, subcellular structural localization, and COG/KOG function.

**Figure 3:**
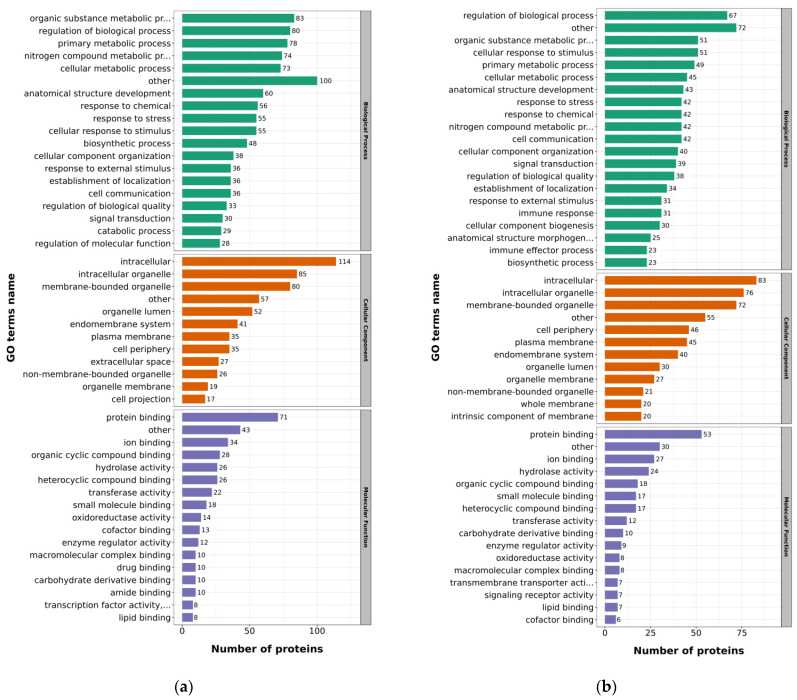
GO annotation of the DEPs. (**a**) GO annotation of the up-regulated proteins; (**b**) GO annotation of the down-regulated proteins.

The identified DEPs were submitted for GO classification to obtain insight into the functional enrichment, including biological processes, cellular components, and molecular functions. The biological process classification results indicated that up-regulated proteins were primarily involved in organic substance metabolic processes, regulation of the distinct biological processes and primary metabolic processes (Figure 3a), whereas most of the down-regulated proteins were involved in regulation of biological processes, organic substance metabolic processes, and cellular response to the stimulus (Figure 3b). The cellular component classification results clearly suggested that both up-regulated and down-regulated proteins primarily originated from intracellular, intracellular organelle and membrane-bounded organelle processes. The molecular function classification results indicated that up-regulated proteins mainly participated in the protein binding, ion binding and organic cyclic compound binding, whereas the down-regulated DEPs mainly participated in protein binding, ion binding and hydrolase activity.

**Figure 4:**
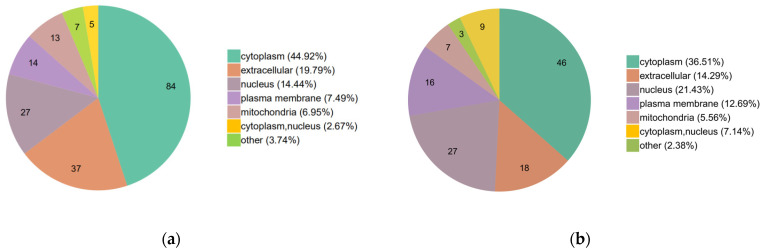
The subcellular localization of the DEPs: (**a**) the subcellular localization of the up-regulated proteins; (**b**) the subcellular localization of the down-regulated proteins.

Further subcellular localization analysis indicated that most of the up-regulated proteins were predominantly localized in the cytoplasm (44.92%), extracellular area (19.79%) and nucleus (14.44%) (Figure 4a), while most of the down-regulated proteins were localized in the cytoplasm (36.51%), nucleus (21.43%) and extracellular area (14.29%) (Figure 4b).

**Figure 5:**
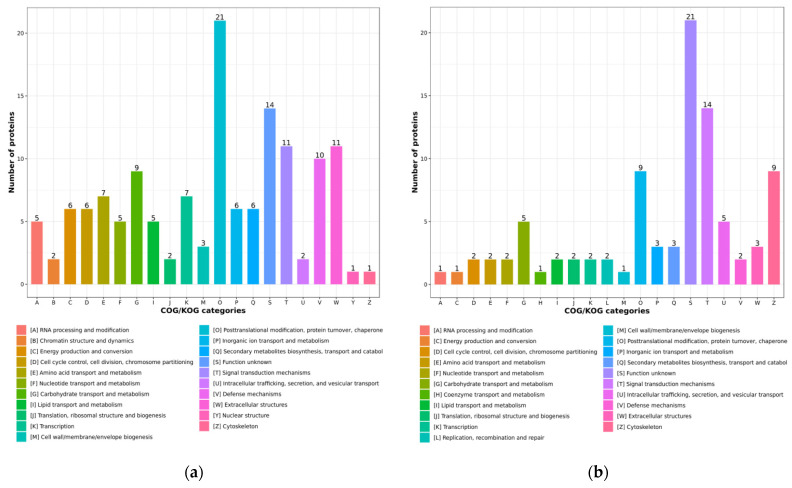
The COG/KOG categories of the DEPs: (**a**) the COG/KOG categories of the up-regulated proteins; (**b**) the COG/KOG categories of the down-regulated proteins.

The Clusters of Orthologous Groups of proteins/Eukaryotic Ortholog Groups (COG/KOG) category analysis results suggested that most of the up-regulated proteins were associated with the posttranslational modifications, protein turnover, chaperones, signal transduction mechanisms and extracellular structures (Figure 5a). On the contrary, most of the down-regulated proteins were linked with the different signal transduction mechanisms, posttranslational modifications, protein turnover, chaperones and cytoskeletons (Figure 5b).

### 2.4. Functional Enrichment of the DEPs

In order to detect whether the DEPs are significantly enriched in certain functional types, GO and KEGG enrichment were analyzed. A negative logarithm (-lg) conversion was performed on the *p*-value obtained by the enrichment test, and the larger the converted value, the more significant this functional type.

**Figure 6:**
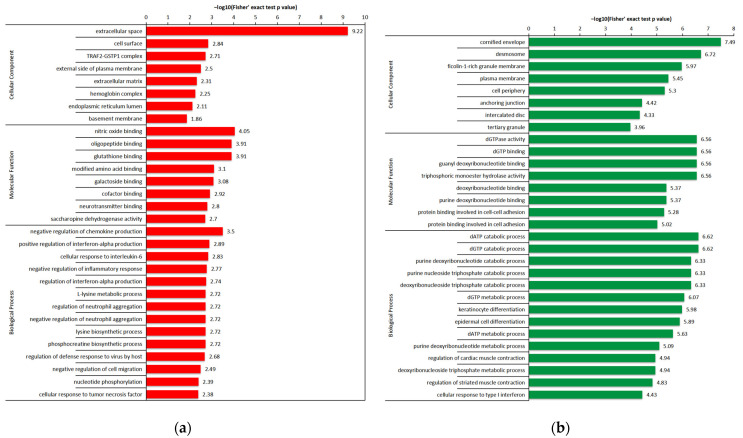
GO enrichment analysis of the DEPs: (**a**) GO enrichment analysis of the up-regulated proteins; (**b**) GO enrichment analysis of the down-regulated proteins.

GO enrichment analyses were performed based on the cellular components, molecular functions and biological processes (Figure 6). According to the cellular components, most of the up-regulated proteins were enriched in the extracellular space, cell surface and TRAF2-GSTP1 complex, whereas the down-regulated proteins were mainly enriched in the cornified envelope, desmosome and ficolin-1-rich granule membrane. According to molecular functions, most up-regulated proteins were enriched in the nitric oxide binding, oligopeptide binding and glutathione binding, whereas the down-regulated proteins were mainly enriched in dGTPase activity, Dgtp binding, guanyl deoxyribonucleotide binding and triphosphoric monoester hydrolase activity. According to the biological processes, most of the up-regulated proteins were mainly enriched in the negative regulation of chemokine production, positive regulation of interferon-alpha production and the cellular response to interleukin-6, whereas the down-regulated proteins were primarily enriched in the Datp catabolic process, Dgtp catabolic process and purine deoxyribonucleotide catabolic process.

**Figure 7:**
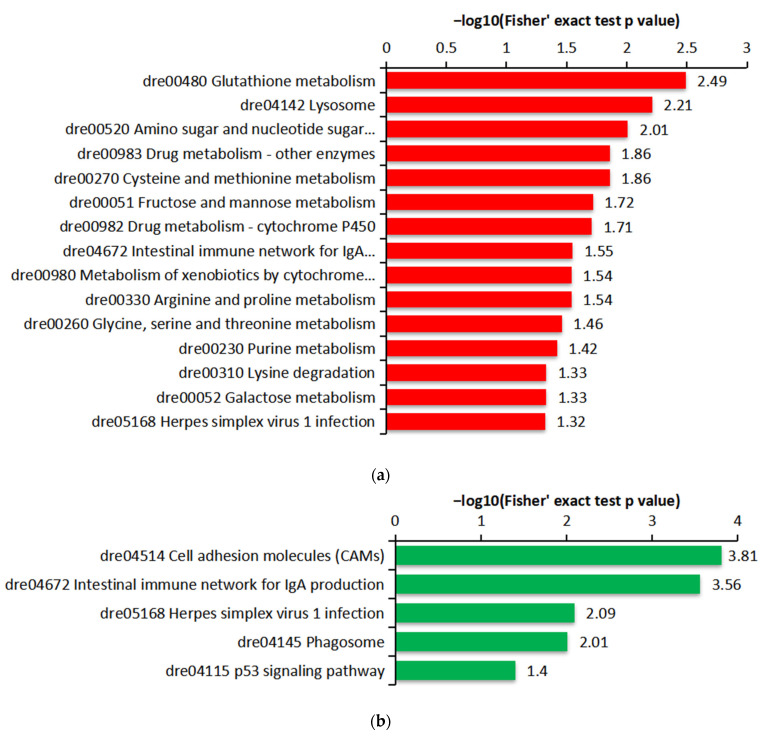
KEGG pathway enrichment analysis of the DEPs: (**a**) KEGG enrichment analysis of the up-regulated proteins; (**b**) KEGG enrichment analysis of the down-regulated proteins.

Furthermore, the Kyoto Encyclopedia of Genes and Genomes (KEGG) pathway enrichment analysis indicated that the various up-regulated proteins were mainly enriched in 15 pathway entries, including the amino acid metabolism, carbohydrate metabolism, lysosome pathway, intestinal immune network pathway, herpes simplex virus 1 infection pathway, etc. (Figure 7a). On the contrary, the down-regulated proteins were mainly enriched in the cell adhesion molecules (CAMs) pathway, intestinal immune network for IgA production pathway, herpes simplex virus 1 infection pathway, phagosome pathway and p53 signaling pathway (Figure 7b).

### 2.5. Confirmation of DEPs by PRM Analysis

To verify the reliability of the TMT-labelled quantitative proteomic analysis results, a total of 19 DEPs were selected for further PRM analysis. A total of 236 fragment ions (including b and y ions) were identified by PRM analysis, and the DEPs detected by PRM were found to be highly consistent with that of TMT (Table 2), which indicated that the TMT data were reliable.

## 3. Discussion

AngHV is an important viral pathogen affecting cultured eels and a major factor leading to the reduction of wild eels. The eel culture industry has developed rapidly in China since the 1990s; however, an epidemic eel disease, “mucus sloughing and hemorrhagic septicemia disease”, has been reported frequently in eel farms. The disease caused by AngHV infection displays typical symptoms of skin mucus sloughing. In this study, quantitative proteomics were used to analyze the skin mucus of AngHV infected eels.

The DEPs were found to be enriched in several pathways. The up-regulated proteins were mainly enriched in the metabolic pathways; however, the down-regulated proteins were all enriched in immune response pathways. These observations indicated that the metabolism of the virus-infected eels was activated, and the immune response was inhibited, which were consistent with the results that herpes virus could cause host immunosuppression [30,31]. In addition, the herpes simplex virus 1 infection pathway, p53 signaling pathway, CAMs pathway and phagosome pathway were also found to be crucial for AngHV infection.

Herpes viruses have a wide range of reservoirs and can infect humans, mammals, birds, and fish. Most herpes viruses tend to invade mucosa tissues of the host. There are also some similarities in the immune response pathways in the host upon their invasion. In the research of the combined transcriptomic/proteomic analysis of *Carassius auratus gibelio* in CyHV-2 infection [29], multiple DEPs were involved in the herpes simplex infection, RIG-I like receptor signaling pathway and p53 signaling pathway. Similar results were achieved in this study. This indicates that DEPs in these pathways might play an important role in immune regulation during the invasion of the host by herpes virus. 

The herpes simplex virus 1 infection pathway is a large complex signaling pathway, which is involved in multiple immune regulatory signaling pathways, including the p53 signaling pathway, RIG-I-like receptor signaling pathway, JAK-STAT signaling pathway, etc. In this study, a variety of immune-related proteins associated with the herpes simplex virus 1 infection pathway was observed to be differentially expressed. These included IRF3, RIG-I, STAT1, CRT, TAP, MHC-I and MHC-II. RIG-I can be activated by invading viral agents and then induce Interferon (IFN) production to resist the viral infection by facilitating phosphorylation of IRF3 [29,32,33,34,35]. We found that the expression of both RIG-I and IRF3 were up-regulated after AngHV infection, which was also found in CyHV-2–infected crucian carp [29] and nervous necrosis virus (NNV)-infected zebrafish [33]. 

IFN plays a key role in the regulation of antiviral innate immune response of the host against virus invasion. STAT1 can be activated and effectively mediate the IFN signaling pathway when the host defense is initiated against viral pathogens [36,37], which can then bind to the cytoplasmic IRF9 to produce the complex ISGF3 (IFN-stimulated gene factor), and finally induce the transcription of ISGs [38]. We found that the expression of STAT1 was substantially upregulated following AngHV infection, thus indicating that the IFN signaling pathway might be activated during AngHV infection. 

MHC is a kind of cell surface–specific transfer protein, which can bind and aid in presenting endogenous and exogenous antigens to T lymphocytes, thus playing an important role in both congenital and acquired immunity. There are three main types of MHC molecules, MHC-I, MHC-II and MHC-III, which can exhibit different functions. MHC-I can interact with CD8+ cells and present endogenous proteins, whereas MHC-II interacts with CD4+ cells and helps in the presentation foreign proteins [39]. Tapasin and CRT can play essential roles in the assembly of MHC-I molecules in ER [40,41]. We found that the expression of tapasin, MHC-I and MHC-II was down-regulated in AngHV-infected eels, which indicated that the MHC-I and MHC-II signaling pathway might be suppressed. 

The findings of this study provide a preliminary and exploratory understanding of the DEPs in the skin mucus of AngHV-infected eels, and more in-depth analysis of the pathways involved in AngHV infection is required to obtain a clearer picture of AngHV invasion mechanisms. 

## 4. Materials and Methods

*Cells and virus*. Eel ovary cells (EO) were cultured with Leibovitz’s L-15 medium (Hyclone) supplemented with 10% fetal bovine serum (FBS, Gibco) and incubated at 27 °C. AngHV strain NA16108 was isolated by our laboratory (Institute of Biotechnology, Fujian Academy of Agricultural Sciences). The viral titers were determined by 50% tissue culture infective dose (TCID_50_) using EO cells.

*Sample collection*. The eel experimental procedures were approved by the Ethics Committee of the Institute of Biotechnology, Fujian Academy of Agricultural Sciences on 21 Aug 2018 (protocol code 2018R-A-3). *Anguilla anguilla* (weight: 10–15 g) were randomly divided into two distinct groups (10 fish/group). Eels in the challenged group were intraperitoneally (i.p.) injected with 5 × 10^6^ PFU of AngHV, whereas eels in control group were injected with EO cell culture medium. At 6 days post-injection, three eels from each group were sedated by administration of tricaine methane sulfonate (TMS), sacrificed and dissected. The skin mucus of the eels was collected and stored at –80 ℃ for further analysis.

*Histopathological analysis*. The skin mucus of the eels was removed and fixed in Bouin’s fixative solution for histopathological analysis. The fixed tissues were paraffin embedded, sectioned, stained with hematoxylin-eosin (HE staining) and observed under a Nikon eclipse E100 microscope. The nucleus was stained with blue and the cytoplasm was stained with red. Nikon DS-U3 imaging software was used for image acquisition and analysis. 

*Protein extraction and digestion*. The skin mucus was collected from each group with 3 replicates and ground with liquid nitrogen and sonicated three times on ice in lysis buffer (8 M urea, 1% Protease Inhibitor Cocktail) using a high intensity ultrasonic processor (Scientz, Ningbo, China). The remaining debris was removed by centrifugation at 12,000× *g* at 4 °C for 10 min, and the supernatant was collected. The protein concentration was thereafter determined using a BCA protein assay kit (Beyotime Biotechnology, Shanghai, China) according to the manufacturer’s instructions. 

The protein solution was reduced with 5 mM dithiothreitol (DTT) for 30 min at 56 °C and alkylated with 11 mM iodoacetamide for 15 min at room temperature in the dark. The protein sample was then diluted by adding 100 mM Triethylamonium bicarbonate (TEAB) buffer to reduce the concentration of urea to less than 2M. Finally, trypsin was added at 1:50 trypsin-to-protein mass ratio for the first round of digestion overnight and 1:100 trypsin-to-protein mass ratios for a second round of 4 h digestion.

*TMT labeling and fractionation*. After the trypsin digestion, peptide was desalted by Strata X C18 SPE column (Phenomenex, Torrance, CA, USA) and vacuum-dried. The peptide was reconstituted in 0.5 M TEAB and processed using a TMT kit (Thermo Fisher Scientific, Waltham, MA, USA) according to the manufacturer’s protocol. Briefly, one unit of TMT reagent was thawed and reconstituted in acetonitrile. The peptide mixtures were then incubated at the room temperature for 2 h and pooled, desalted and dried by vacuum centrifugation. Thereafter, the tryptic peptides were fractionated by high pH reverse-phase HPLC using Agilent 300Extend-C18 column (5 μm particles, 4.6 mm i.d., 250 mm length). Briefly, peptides were first separated with a gradient of 8% to 32% acetonitrile (pH 9.0) over 60 min into 60 fractions. Finally, the peptides were combined into 14 different fractions and dried by vacuum centrifugation.

*LC-MS/MSanalysis*. Detailed MS setting parameters are listed in Table S4. Briefly, the peptide fractions were dissolved in buffer A (0.1% formic acidin 2% acetonitrile) and then separated with an increased linear gradient of buffer B (0.1% formic acid in 90% acetonitrile), which was increased from 7% to 22% for 26 min, 22% to 32% for 8 min and then increased to 80% for 3 min. It was then held constant at 80% for the last 3 min, all at a constant flow rate of 400 nL/min on an EASY-nLC 1000 UPLC system. Then, the peptides were subjected to an NSI source followed by tandem mass spectrometry (MS/MS) in Q Exactive^TM^ Plus (Thermo Fisher Scientific) coupled online to EASY-nLC 1000 UPLC at 2.0 kV electrospray voltage. The m/z scan range was 400 to 1500 for a full scan, and intact peptides were detected in the Orbitrap at a resolution of 60,000. Peptides were then selected for MS/MS using the NCE setting of 100, and the fragments were detected in the Orbitrap at a resolution of 15,000. A data-dependent procedure alternated between one MS scan followed by 20 MS/MS scans with 30.0 s dynamic exclusion. Automatic gain control (AGC) was set at 5E4. Fixed first mass was set as 70 m/z. 

*Database search and protein identification*. Detailed peptide identification parameters are listed in Table S4. The resulting MS/MS data were processed using Maxquant search engine (v.1.5.2.8). The tandem mass spectra were searched against proteins of *Anguilla sp.* (Organism_ID: 7935) and *Danio rerio* (Organism_ID: 7955) in the UniProt database, downloaded on 20 May 2019, concatenated with the reverse decoy database. Trypsin/P was specified as the cleavage enzyme, allowing up to 2 missed cleavages. The minimum length of the peptide was set to 7 amino acid residues. The maximum number of peptide modifications was set to 5. The mass tolerance for the precursor ions was set as 20 ppm in the first search and 5 ppm in the main search, and the mass tolerance for fragment ions was set as 0.02 Da. Carbamidomethyl on Cys was specified as the fixed modification. Oxidationson Met, Acetyl (Protein N-term) and NQ were specified as variable modifications. The quantitative method was set to TMT-6PLEX, and the false discovery rate (FDR) was adjusted to < 1%.

*Identification of the DEPs.* The fold change (*FC*) was calculated by the ratio of the mean intensity for each protein in two sample groups. The student’s t test was performed on the relative quantitative value of each protein from the two sample groups. The corresponding *p*-value was calculated as the significance index, and a *p*-value less than 0.05 was regarded as DEP. The relative quantitative value of proteins was applied with log2 transformation. 

*Bioinformatics analysis*. GO annotations were derived from the UniProt-GOA database (http://www.ebi.ac.uk/GOA/, accessed on 22 August 2019) and complemented with InterProScan software. The DEPs were then classified into the different biological processes, cellular components and molecular functions, and their subcellular localization was predicted using WoLF PSORT software. The annotated proteins were then matched into the corresponding pathways using the KEGG database. Enrichment analysis of the identified proteins was performed using two-tailed Fisher’s exact test for each category to analyze the functional enrichment or depletion of the identified proteins compared with all of the proteins present in the database. The terms with *p*-values of less than 0.05 were considered as significant. 

*Parallel reaction monitoring (PRM) analysis*. A total of 19 proteins, including 8 up-regulated proteins and 11 down-regulated proteins, involving virus infection and host immune response were selected for further PRM analysis (Table S3). The same batch of skin mucus samples were used for further PRM analysis to verify the results obtained from TMT analysis. Briefly, protein extraction and trypsin digestion methods were performed as described in the TMT experiment. Then, the peptide fractions were dissolved in buffer A (0.1% formic acid in 2% acetonitrile) and then separated with an increased linear gradient of buffer B (0.1% formic acid in 90% acetonitrile), which was increased from 6% to 25% for 16 min, 25% to 35% for 6 min and then increased to 80% for 4 min. It was then held constant at 80% for the last 4 min, all at a constant flow rate of 500 nL/min on an EASY-nLC 1000 UPLC system. The peptides were subjected to an NSI source followed by MS/MS in Q Exactive^TM^ Plus (Thermo) coupled online to the UPLC. The electrospray voltage applied was 2.1 kV. The m/z scan range was 325 to 1190 for the full scan, and intact peptides were detected in the Orbitrap at a resolution of 70,000. Peptides were then selected for MS/MS using the NCE setting of 27, and the fragments were detected in the Orbitrap at a resolution of 17,500. A data-independent procedure alternated between one MS scan followed by 20 MS/MS scans. Automatic gain control (AGC) was set at 3E6 for full MS and 1E5 for MS/MS. The maximum IT was set at 50 ms for full MS and 110 ms for MS/MS. The isolation window for MS/MS was set at 1.6 m/z. The peptide settings used were as follows: the enzyme was set as trypsin [KR/P], the max number of missed cleavages as 0, the peptide length was set as 7–25, the fixed modifications as alkylation on Cys. For the transition settings, precursor charges were set as 2, 3; the ion charges as 1; and ion types as b, y. Moreover, the productions were set from ion 3 to the last ion, and the ion match tolerance as 0.02 Da.

## 5. Conclusions

In conclusion, a total of 187 up-regulated proteins and 126 down-regulated proteins were found in the skin mucus of the eels infected with AngHV. Most of the identified DEPs were enriched in the CAMs pathway, intestinal immune pathway, herpes simplex virus 1 infection pathway, phagosome pathway and p53 signaling pathway. Overall, the results provided important information for the elucidation of pathogenesis of AngHV and understanding the various defense mechanisms of infected eels.

## Figures and Tables

**Table 1:** Summary of MS/MS spectrum database search analysis.

Total Spectrum	Matched Spectrum	Peptides	Unique Peptides	Identified Proteins	Quantified Proteins
298,207	44,795 (15.0%)	20,633	16,815	3486	2935

**Table 2:** The result of the DEP detection by PRM.

Protein Accession	Protein Name	Protein Description	Fold Change (AngHV/CK)	
			PRM	TMT
transcript_14379	STAT1	signal transducer and activator of transcription 1	1.888 ± 0.021	1.915 ± 0.040
transcript_28497	CRISP-3	Cysteine-rich secretory protein 2	1.847 ± 0.085	1.698 ± 0.028
transcript_25607	serpin B	serpin B	1.695 ± 0.066	1.788 ± 0.024
transcript_15352	SAP	saposin	1.406 ± 0.092	1.312 ± 0.029
transcript_15360	CD68 antigen	CD68 antigen	1.372 ± 0.007	1.603 ± 0.006
transcript_25331	cathepsin B	cathepsin B [EC:3.4.22.1]	1.354 ± 0.070	1.325 ± 0.036
transcript_29917	HBA	hemoglobin subunit alpha	1.352 ± 0.132	1.585 ± 0.091
transcript_29975	HBE	hemoglobin subunit epsilon	1.191 ± 0.126	1.567 ± 0.079
transcript_1706	DSG2	desmoglein 2	0.683 ± 0.024	0.767 ± 0.003
transcript_23496	CAV-1	caveolin 1	0.668 ± 0.041	0.758 ± 0.013
transcript_27261	ASC	apoptosis-associated speck-like protein containing a CARD	0.622 ± 0.009	0.707 ± 0.012
transcript_7859	MTHFD1	methylenetetrahydrofolate dehydrogenase (NADP+)/methenyltetrahydrofolate cyclohydrolase/formyltetrahydrofolate synthetase [EC:1.5.1.5 3.5.4.9 6.3.4.3]	0.551 ± 0.017	0.765 ± 0.022
transcript_2511	plakophilin 1	plakophilin 1	0.511 ± 0.028	0.712 ± 0.006
transcript_8206	CTNNβ1	catenin beta 1	0.451 ± 0.007	0.678 ± 0.007
transcript_3978	ADA	adenosine deaminase [EC:3.5.4.4]	0.364 ± 0.014	0.463 ± 0.007
transcript_1362	desmocollin 2	desmocollin 2	0.306 ± 0.024	0.527 ± 0.007
transcript_893	DSP	desmoplakin	0.276 ± 0.010	0.052 ± 0.002
transcript_9314	SAMHD1	deoxynucleoside triphosphate triphosphohydrolase SAMHD1	0.027 ± 0.004	0.130 ± 0.006
transcript_24094	MHC II	major histocompatibility complex, class II	0.007 ± 0.001	0.445 ± 0.015

## Data Availability

The mass spectrometry proteomics data from TMT labeling analysis and PRM analysis were deposited to the ProteomeXchange Consortium via the PRIDE partner repository with the dataset identifier PXD034867.

## Supplementary Materials

The following supporting information can be downloaded at: https://www.mdpi.com/article/10.3390/ijms231911283/s1.
