# Supplementary material for: Proteomic Profiling Skin Mucus of European Eel Anguilla anguilla Infected with Anguillid Herpesvirus
Source: Int J Mol Sci. 2022 Sep 24;23(19):11283. doi: 10.3390/ijms231911283 (PMC9570476; doi:10.3390/ijms231911283)
Supplement: Supplementary file 1 [file ijms-23-11283-s001.zip › Figure S1.pdf]

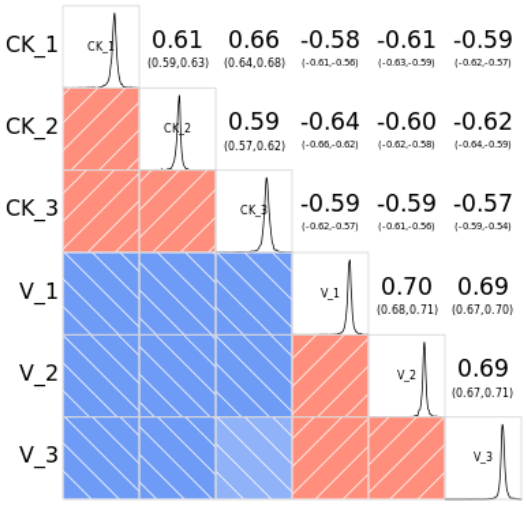

Figure S1. The heat map of the Pearson correlation coefficient for protein quantification between pairwise samples. The closer Pearson's coefficient is to -1, the negative correlation; the closer Pearson's coefficient is to 1, the positive correlation; and the closer Pearson's coefficient is to 0, the uncorrelation.
