# Supplementary material for: Proteomic Profiling Skin Mucus of European Eel Anguilla anguilla Infected with Anguillid Herpesvirus
Source: Int J Mol Sci. 2022 Sep 24;23(19):11283. doi: 10.3390/ijms231911283 (PMC9570476; doi:10.3390/ijms231911283)
Supplement: Supplementary file 1 [file ijms-23-11283-s001.zip › Table S3.pdf]

Table S3. The target proteins selected for PRM analysis

Protein Accession	Protein Description	Regulated Type	V/CK *p* Value
F02_transcript_14379	signal transducer and activator of transcription 1-like; K11220 signal transducer and activator of transcription 1 (A)	Up	0.00045973
F02_transcript_28497	Cysteine-rich secretory protein 2 [Zootermopsis nevadensis]	Up	0.0029971
F02_transcript_25607	serpinb14, im:7148243, si:ch73-303g23.4; serpin peptidase inhibitor, clade B (ovalbumin), member 14; K13963 serpin B (A)	Up	0.000122942
F02_transcript_15352	proactivator polypeptide-like; K12382 saposin (A)	Up	0.0028559
F02_transcript_15360	lysosome-associated membrane glycoprotein 1-like; K06501 CD68 antigen (A)	Up	0.0064393
F02_transcript_25331	ctsba, ctsb, id:ibd1201, wu:fa13g05, wu:fb34e12, zgc:55862, zgc:65809, zgc:77181; cathepsin Ba (EC:3.4.22.1); K01363 cathepsin B [EC:3.4.22.1] (A)	Up	0.023523
F02_transcript_29917	hemoglobin subunit alpha-A-like; K13822 hemoglobin subunit alpha (A)	Up	0.0153992
F02_transcript_29975	ba1l, si:bY187G17.4; ba1 globin, like; K13825 hemoglobin subunit epsilon (A)	Up	0.0142045
F02_transcript_1706	desmoglein-2-like; K07597 desmoglein 2 (A)	Down	7.8735E-05
F02_transcript_23496	caveolin-1-like; K06278 caveolin 1 (A)	Down	0.0061574
F02_transcript_27261	PYCARD; PYD and CARD domain containing; K12799 apoptosis-associated speck-like protein containing a CARD (A)	Down	0.00092485
F02_transcript_7859	c-1-tetrahydrofolate synthase, cytoplasmic-like; K00288 methylenetetrahydrofolate dehydrogenase (NADP+)/ methenyltetrahydrofolate cyclohydrolase / formyltetrahydrofolate synthetase [EC:1.5.1.5 3.5.4.9 6.3.4.3] (A)	Down	0.0094153
F02_transcript_2511	plakophilin-1-like; K10387 plakophilin 1 (A)	Down	5.5521E-05
F02_transcript_8206	catenin beta-1-like; K02105 catenin beta 1 (A)	Down	0.000122257
F02_transcript_3978	adenosine deaminase-like; K01488 adenosine deaminase [EC:3.5.4.4] (A)	Down	0.00033975
F02_transcript_1362	dsc2l, dsc, sb:cb859, wu:fa94h05; desmocollin 2 like; K07601 desmocollin 2 (A)	Down	5.545E-05
F02_transcript_893	dspa, im:6911953, sb:cb570, wu:fc17a08, wu:fk73d01; desmoplakin a; K10381 desmoplakin (A)	Down	0.000584
F02_transcript_9314	deoxynucleoside triphosphate triphosphohydrolase SAMHD1 [Gallus gallus]	Down	1.79666E-05
F02_transcript_24094	zgc:158870; K06752 major histocompatibility complex, class II (A)	Down	0.00026136
