# Supplementary material for: Proteomic Profiling Skin Mucus of European Eel Anguilla anguilla Infected with Anguillid Herpesvirus
Source: Int J Mol Sci. 2022 Sep 24;23(19):11283. doi: 10.3390/ijms231911283 (PMC9570476; doi:10.3390/ijms231911283)
Supplement: Supplementary file 1 [file ijms-23-11283-s001.zip › Table S4.pdf]

Table S4. The parameters used for mass spectrum analysis and peptides identification.

Mass Spectrum Parameters				The Peptides Parameters	
**Name**	**Setting**	**Name**	**Setting**	Name	**Setting**
Use lock masses	No	Fixed first mass	100	Enzyme digestion method	Trypsin/P
Peptide match	Yes	NCE/stepped NCE	32	Number of missed cuts	2
MS AGC target	2.00E+05	Isolation window	1.6	mass tolerance for the precursor ion	10.0 ppm
MS Maximum IT	50 ms	Intensity threshold	2.00E+04	mass tolerance for fragment ion	0.02 Da
MS Scan range	400-1500	Charge exclusion	1, ≥6	fixed modification	Carbamidomethyl(C)
MS Resolution	60,000	ddMS2 TopN	20	variable modification	Oxidation(M), Acetyl (Protein N-term), NQ
MS2 Resolution	15,000	MS2 AGC target	5.00E+04	quantitative method	TMT 6plex
Dynamic exclusion	30s	MS2 Maximum IT	70 ms	Peptide scoring threshold	Aug-40
